# Thermal sensitivity and flexibility of the Cε3 domains in immunoglobulin E

**DOI:** 10.1016/j.bbapap.2017.08.005

**Published:** 2017-11

**Authors:** Katy A. Doré, Anna M. Davies, Nyssa Drinkwater, Andrew J. Beavil, James M. McDonnell, Brian J. Sutton

**Affiliations:** King's College London, Randall Division of Cell and Molecular Biophysics, New Hunt's House, London SE1 1UL, United Kingdom; Medical Research Council & Asthma UK Centre in Allergic Mechanisms of Asthma, London, United Kingdom

**Keywords:** Fcε3–4, sub-fragment of IgE-Fc consisting of the dimer of Cε3 and Cε4 domains, DSF, differential scanning fluorimetry, GlcNAc, *N*-acetylglucosamine, Man, mannose, Antibody, Immunoglobulin E, Glycosylation, Domain flexibility, Thermal unfolding, Differential scanning fluorimetry

## Abstract

Immunoglobulin E (IgE) is the antibody that plays a central role in the mechanisms of allergic diseases such as asthma. Interactions with its receptors, FcεRI on mast cells and CD23 on B cells, are mediated by the Fc region, a dimer of the Cε2, Cε3 and Cε4 domains. A sub-fragment lacking the Cε2 domains, Fcε3–4, also binds to both receptors, although receptor binding almost exclusively involves the Cε3 domains. This domain also contains the N-linked glycosylation site conserved in other isotypes. We report here the crystal structures of IgE-Fc and Fcε3–4 at the highest resolutions yet determined, 1.75 Å and 2.0 Å respectively, revealing unprecedented detail regarding the carbohydrate and its interactions with protein domains. Analysis of the crystallographic B-factors of these, together with all earlier IgE-Fc and Fcε3–4 structures, shows that the Cε3 domains exhibit the greatest intrinsic flexibility and quaternary structural variation within IgE-Fc. Intriguingly, both well-ordered carbohydrate and disordered polypeptide can be seen within the same Cε3 domain. A simplified method for comparing the quaternary structures of the Cε3 domains in free and receptor-bound IgE-Fc structures is presented, which clearly delineates the FcεRI and CD23 bound states. Importantly, differential scanning fluorimetric analysis of IgE-Fc and Fcε3–4 identifies Cε3 as the domain most susceptible to thermally-induced unfolding, and responsible for the characteristically low melting temperature of IgE.

## Highlights

• The Cε3 domains of IgE are most susceptible to thermally induced unfolding determined by differential scanning fluorimetry.
• The Cε3 domains are responsible for the characteristically low melting temperature of IgE.
• The Cε3 domains exhibit the greatest intrinsic flexibility.
• Quaternary structural diversity of Cε3 domains is compared across all known structures using a simplified single parameter.
• Human IgE-Fc and Fcε3–4 domain structures are determined at the highest resolutions yet reported (1.75 Å and 2.0 Å).

## 1 Introduction

IgE plays a central role in the molecular and cellular mechanisms of allergy, and is a validated therapeutic target in the development of new approaches to treat allergic diseases such as asthma. It exerts its effects through its two principal receptors, FcεRI on mast cells, basophils and antigen-presenting cells, and CD23/FcεRII on B cells. The former mediates allergic hypersensitivity, the latter regulates IgE levels, and both contribute to allergen presentation to the immune system [1], [2].

IgE causes long term sensitisation of mast cells due to the uniquely slow off-rate for the antibody/receptor complex (*k*_*off*_ = 1.9 × 10^− 4^ s^− 1^), and this is reproduced with the IgE-Fc fragment containing the dimer of Cε2, Cε3 and Cε4 domains [3]. The FcεRI binding site comprises two sub-sites, one on each Cε3 domain [4], [5], and although the Cε2 domains are not directly involved, they contribute to this slow off-rate since the Fcε3–4 fragment, lacking these domains, has a somewhat faster off-rate (*k*_*off*_ = 3.2 × 10^− 3^ s^− 1^) [3]. The overall affinities of IgE-Fc and Fcε3–4 for FcεRI are, however, comparable and high (K_a_ ≈ 10^10^ M^− 1^). The CD23 binding site lies principally within the Cε3 domain but with a small contribution from Cε4 [6]; the affinity of both IgE-Fc and Fcε3–4 for CD23 is lower (K_a_ ≈ 10^7^ M^− 1^), with one binding site in each chain. The Cε3 domain is thus the critical domain for receptor binding.

When expressed alone, and in contrast to Cε2 and Cε4, the Cε3 domain displays a poorly ordered, “molten globule” structure [7], [8], [9], [10]. However, this isolated domain binds, albeit with lower affinity, to sFcεRIα [7], [11], in the process becoming more ordered [8], [9]. This is also the domain to which N-linked carbohydrate is attached, at a site that is conserved across other immunoglobulin isotypes; in IgE the glycosylation is of the high-mannose type [12], [13], [14], [15].

There are now several crystal structures of both IgE-Fc and Fcε3–4 alone and in complex with soluble IgE-binding extracellular domains of FcεRI (sFcεRIα), CD23 (sCD23) and various inhibitors. The first crystal structure was of Fcε3–4 bound to sFcεRIα (1F6A) [4], and this was followed by that of the free Fcε3–4 (1FP5) [16]. The structure of IgE-Fc revealed the organization of the Cε2 domains for the first time (1O0V) [17], and caused much surprise when they were observed bent back onto the Fcε3–4 domains in an asymmetric manner. Three new crystal forms of Fcε3–4 were then determined (3H9Y, 3H9Z, 3HA0) [18], which revealed conformational flexibility between the structures. IgE-Fc bound to sFcεRIα (2Y7Q) was determined along with a higher resolution structure of the free IgE-Fc (2WQR) [5]. The structure of Fcε3–4 with an engineered disulphide bond, trapping Fcε3–4 in an artificially closed, non-receptor binding conformation was determined (4GT7) [19] and the structure of the same constrained form of Fcε3–4 was determined bound by two DARPin (designed ankyrin repeat protein) molecules (4GRG) [20]. The structure of Fcε3–4 bound to calcium-free sCD23 was then determined (4EZM) [6] and subsequently the calcium-bound structure was also solved (4GKO) [21], as well as another crystal form of Fcε3–4 bound to sCD23 (4KI1) [22]. IgE-Fc bound by two anti-IgE Fabs (aεFabs) (4J4P) [23] unexpectedly captured the Fc in a fully extended conformation. The crystal structure of Fcε3–4 bound by the neutralizing antibody Fab MEDI4212 was also determined (5ANM) [24]. The complexes of two anti-IgE omalizumab Fabs bound to the artificially constrained Fcε3–4 (5HYS) [25] and to the native IgE-Fc (5G64) [26] were recently reported. The structure of the asymmetric complex of IgE-Fc bound to a single sCD23 molecule was also recently solved (5LGK) [27].

In FcεRI-bound structures, the Cε3 domains move apart into an “open” conformation to accommodate receptor binding, compared with the uncomplexed fragments. However, binding to CD23 between the Cε3 and Cε4 domains causes the Cε3 domains to adopt a more “closed” conformation, and in this way the binding to the two receptors, while occurring at distant sites at either end of the Cε3 domains, is mutually exclusive [6], [28].

The thermal lability of IgE has been recognised for a long time. Even before the recognition of IgE as an antibody, the loss of “reaginic” activity in atopic sera by heating to 56 °C was reported [29], and shortly after the discovery of the IgE antibody class, this unusually low thermal susceptibility compared with IgG (for which T_m_ values are typically ~ 72 °C), was confirmed [30].

Here we report two new crystal forms of Fcε3–4 (at 2.26 Å and 2.2 Å resolution) together with one of the earlier forms now solved at the highest resolution yet reported (2.0 Å) and IgE-Fc also solved at the highest resolution yet reported (1.75 Å); new details concerning carbohydrate/protein interactions are revealed. We compare these with all previous structures, and propose a simplified, single-parameter descriptor for the quaternary structure of the Cε3 and Cε4 domains. An analysis of B-factors for all IgE-Fc and Fcε3–4 structures is also reported, and correlated with experimental data from differential scanning fluorimetry (DSF), which reveals the Cε3 domains to be responsible for the thermal sensitivity of IgE.

## 2 Materials and methods

### 2.1 Protein preparation and crystallisation

IgE-Fc and Fcε3–4 were prepared as reported earlier [6], [31], and were a kind gift from Dr. A.J. Henry (UCB Pharma, Slough, UK). Crystals were grown at 18 °C using the sitting drop vapour diffusion method. Fcε3–4 crystals in the P1 and P2_1_ ‘large’ form were grown in 0.1 M SPG (succinic acid, sodium dihydrogen phosphate and glycine in the ratio 2:7:7) at pH 6.0 (Hampton Research) and 25% (w/v) PEG1500 using a reservoir volume of 70 μL and drops comprising 100 nL protein solution (10 mg/mL) and 100 nL reservoir. The Fcε3–4 P2_1_ ‘small’ form was grown in 0.1 M MES at pH 5.5, 25% (w/v) PEG 4000 (Hampton Research) and 0.15 M ammonium sulphate using a reservoir volume of 80 μL and drops comprising 100 nL protein solution (7.7 mg/mL) and 200 nL reservoir. IgE-Fc crystals were grown in 0.1 M Tris HCl at pH 8.5 and 25% (v/v) PEG 550 MME (Hampton Research), using a reservoir volume of 80 μL and drops comprising 250 nL protein solution (6 mg/mL) and 250 nL reservoir.

Crystals were cryoprotected as follows: Fcε3–4 P1 crystals were cryoprotected in 0.1 M SPG at pH 4.4 and 30% (w/v) PEG 1500, Fcε3–4 P2_1_ ‘large’ crystals in 0.1 M sodium di‑hydrogen phosphate at pH 4.0 and 30% (w/v) PEG 3350, Fcε3–4 P2_1_ ‘small’ crystals in 25% (w/v) PEG 4000 and 18% (v/v) PEG 400, and IgE-Fc crystals in 0.1 M Tris-HCl at pH 8.5, 25% (v/v) PEG 550 MME and 10% (v/v) PEG 400, before flash-cooling in liquid nitrogen.

### 2.2 Structure determination, model building and refinement

Data were collected at the Diamond Light Source (Harwell, UK). For the Fcε3–4 P1 crystals (beamline I02), data were collected using an ADSC Q315r detector (250 images, 1° oscillation, 0.7 s exposure, λ 0.9795 Å). For the Fcε3–4 P2_1_ ‘large’ crystal form (beamline I04), data were collected using an ADSC Q315r detector (235 images, 0.7° oscillation, 0.87 s exposure; λ 0.9763 Å). For the Fcε3–4 P2_1_ ‘small’ crystal form (beamline I02) data were collected using a Pilatus 6M-F detector (1600 images, 0.2° oscillation, 0.2 s exposure, λ 0.97949 Å). For the IgE-Fc crystal (beamline I04-1), data were collected using a Pilatus 6M-F detector (1800 images, 0.2° oscillation, 0.2 s exposure, λ 0.92819 Å). Data were integrated with DIALS using the Xia2 package [32] and scaled with AIMLESS [33] from the CCP4 suite [34].

The number of molecules in each asymmetric unit (Table 1) was correctly predicted using Matthews probability analysis [35], [36]. All structures were solved by molecular replacement with PHASER [37] using the Fcε3–4 structures, PDB entries 3HA0, 3H9Y and 3H9Z [18], as search models for the P1 and P2_1_ ‘large’ and P2_1_ ‘small’ structures, respectively. For the IgE-Fc structure, PDB entry 2WQR [5] was used as a search model. The Fcε3–4 P2_1_ ‘large’ structure is the same crystal form as 3H9Y, and the IgE-Fc structure is the same crystal form as 2WQR, but both structures represent an improvement in terms of resolution. Refinement was carried out in iterative cycles with PHENIX [38] using weight optimization, and alternated with manual model building using COOT [39]. Structure quality was assessed with MolProbity [40]. Data processing and refinement statistics for all structures are presented in Table 1. Figures were prepared with PyMOL (Version 1.8.2.1 Schrödinger, LLC).

**Table 1:** Data processing and refinement statistics.

Data processing	Fce3–4 (1)	Fe3–4 (2)	Fce3–4 (3)	IgE-Fc
Structure name	P 1	P 2_1_ “large”	P 2_1_ “small”	IgE-Fc
No. of molecules in asymmetric unit	3	2	1	1
Space group	P 1	P 1 2_1_ 1	P 1 2_1_ 1	P 2_1_ 2_1_ 2
Unit cell dimensions (Å)	*a* = 47.67	*a* = 66.48	*a* = 46.53	*a* = 130.67
	*b* = 90.30	*b* = 100.36	*b* = 104.07	*b* = 75.78
	*c* = 92.91	*c* = 77.86	*c* = 52.94	*c* = 79.82
	a = 114.41°	ß = 97.35°	ß = 101.54°	
	ß = 90.63°			
	? = 96.10°			
Resolution (Å): overall (outer shell)	81.61–2.20	100.36–2.00	51.87–2.26	79.82–1.75
	(2.25–2.20)	(2.05–2.00)	(2.33–2.26)	(1.80–1.75)
Completeness (%)^a^	98.9 (97.0)	100 (100)	99.9 (99.9)	99.9 (99.8)
Multiplicity^a^	5.7 (3.8)	14 (8.8)	4.1 (4.2)	13.1 (11.4)
Mean ((I)/s(I))^a^	7.5 (2.0)	14 (1.6)	5.9 (2.0)	14.0 (1.3)
R_merge_^a^	0.13 (1.103)	0.291 (2.218)	0.130 (1.834)	0.098 (1.982)
R_pim_^a^	0.085 (0.899)	0.069 (1.143)	0.069 (1.000)	0.028 (0.607)
CC_1/2_^a^	0.997 (0.656)	0.995 (0.651)	0.993 (0.362)	0.999 (0.495)
Wilson *B*-factor (Å^2^)	43.66	32.92	46.51	25.10

Refinement	Fce3–4 (1)	Fe3–4 (2)	Fce3–4 (3)	IgE-Fc

R_work_/R_free_ (%)^b^	21.34/23.36	20.24/22.60	20.15/23.56	19.73/23.23
No. of reflections	60,090	68,373	23,108	80,159
RMSD:				
Bond lengths (Å)	0.003	0.003	0.006	0.009
Bond angles (°)	0.591	0.619	0.767	0.944
Coordinate error (Å)	0.27	0.25	0.30	0.27
No. of atoms:				
Protein	8732	6358	2986	4933
Carbohydrate	346	263	122	144
Solvent	213	519	37	601
Other	73^c^	33^d^	50^e^	72^f^
Ramachandran plot:				
Favoured (%)	98.09	98.28	97.42	98.00
Allowed (%)	99.82	100	100	100

a Values in parentheses are for the highest resolution shell.

b R_free_ set comprises 5% of reflections.

c Ethylene glycol, polyethylene glycol, phosphate.

d Ethylene glycol, polyethylene glycol.

e Ethylene glycol, polyethylene glycol, sulphate.

f Ethylene glycol, polyethylene glycol.

In the IgE-Fc structure, residues were built as follows: chain A 228–544; chain B 225–545. In the Fcε3–4 P2_1_ ‘small’ structure: chain A 336–365, 370–424, 427–544; chain B 336–362, 369–422, 429–544. In the Fcε3–4 P2_1_ ‘large’ structure: chain A 336–424, 428–546; chain B 336–363, 369–545; chain C 335–362, 369–421, 428–544; chain D 336–366, 369–422, 428–544. In the Fcε3–4 P1 structure: chain A 339–360, 372–389, 398–419, 430–544; chain B 338–362, 369–393, 398–421, 428–544; chain C 336–544; chain D 335–362, 369–421, 429–498, 503–544; chain E 336–362, 369–423, 425–454, 457–544; chain F: 339–361, 372–376, 379–381, 383–389, 394–418, 431–454, 456–544. Carbohydrate was built at Asn394 as follows: IgE-Fc structure chain A GlcNAc_2_Man_3_; chain B GlcNAc_2_Man_5_. In the Fcε3–4 P2_1_ ‘small’ structure: chain A GlcNAc_2_Man_3_; chain B GlcNAc_2_Man_3_. In the Fcε3–4 P2_1_ ‘large’ structure: chain A GlcNAc_2_Man_2_; chain B GlcNAc_2_Man_5_; chain C GlcNAc_1_Man_3_; chain D GlcNAc _2_Man_5_. In the Fcε3–4 P1 structure: chain A GlcNAc_1_Man_3_; chain B GlcNAc_2_Man_3_; chain C GlcNAc_2_Man_3_; chain D GlcNAc_2_Man_5_; chain E GlcNAc_2_Man_3_; chain F GlcNAc_0_Man_3_. For the quaternary structural comparisons, the angle defined by the three Cα atoms X337-X497-Y337 or Y337-Y497-X337 (where X and Y refer to two paired chains) was measured using the PyMOL Molecular Graphics System (Version 1.8.2.1 Schrödinger, LLC). NB: where residues 337 or 497 were not modelled in the structure (Fcε3–4 P1 structure chains A and B, structure 5ANM chain A, B, C and D and structure 3H9Y chain A, B and C), the measurement was not taken.

### 2.3 B-factor analyses of structures

All chains of the four new structures presented here, together with all published Fcε3–4 and IgE-Fc structures, were analysed. To compare B-factors derived from different structure determinations, each was normalised by division by the average B-factor for the whole Cε3–4 chain (for comparison of Cε3 and Cε4) or Cε3 domain (for comparison of the Cε4-distal region and Cε4-proximal region). The Cε3/Cε4 boundary was defined as residues 437/438; the Cε4-distal region of Cε3 was defined as residues 336–339, 359–372, 387–400 and 419–431; the Cε4-proximal region of Cε3 was defined as residues 340–358, 373–386, 401–418 and 432–437.

### 2.4 Differential scanning fluorimetry

Thermal stability assays were carried out according to a published protocol [41] using the Stratagene Mx3005p RT-PCR instrument. Sypro orange was used as the fluorescent dye (Life Technologies) with filters at 492 nm (excitation) and 610 nm (emission). The concentration for both proteins was 5μM. Each sample was measured in duplicate for Fcε3–4 and in triplicate for IgE-Fc. Positive controls were performed with lysozyme (data not shown) and negative controls as follows: protein with no Sypro orange, Sypro orange with buffer, and buffer alone (data not shown). Melting temperatures were taken as the maxima of the first derivative calculated using Graphpad Prism version 7 (Table 2).

**Table 2:** Melting temperatures for Fcε3–4 and IgE-Fc.

Urea concentration (M)	T_m_ (Fcε3–4) (°C)	T_m_1 (IgE-Fc) (°C)	T_m_2 (IgE-Fc) (°C)
0	52	55	64
1	48	50	63
2	47	45	57
3	39	39	53
4	–	–	49
5	–	–	46
6	–	–	42

### 2.5 Mass spectrometry

The two protein samples, either IgE-Fc or Fcε3–4, were dialysed into a solution of H_2_O, 10% acetonitrile and 0.1% formic acid prior to mass spectrometric analyses. Samples at concentrations between 8 μg/mL and 80 μg/mL were directly infused at 25 μL/min into a Bruker MaXis ultrahigh-resolution electrospray ionization quadrupole time-of-flight mass spectrometer. The multiple charge states were deconvoluted using the manufacturer's maximum entropy algorithm (Bruker Daltonics). Due to glycosylation heterogeneity, a series of masses were observed with well-defined mass differences of 162 Da, corresponding to variations in the number of mannose residues present in the sample [12]. For IgE-Fc, the derived masses were consistent with intact protein with a single N-linked glycosylation site per chain, with two GlcNAc residues and 1–7 mannose residues. For Fcε3–4, the derived masses were consistent with intact protein with a single N-linked glycosylation site per chain, containing two GlcNAc residues and 5–9 mannose residues. Both protein samples also show evidence of partial loss of their C-terminal lysine residue, a common modification observed in recombinantly expressed antibodies [42].

## 3 Results and discussion

### 3.1 Overall structure

**Fig. 1:**
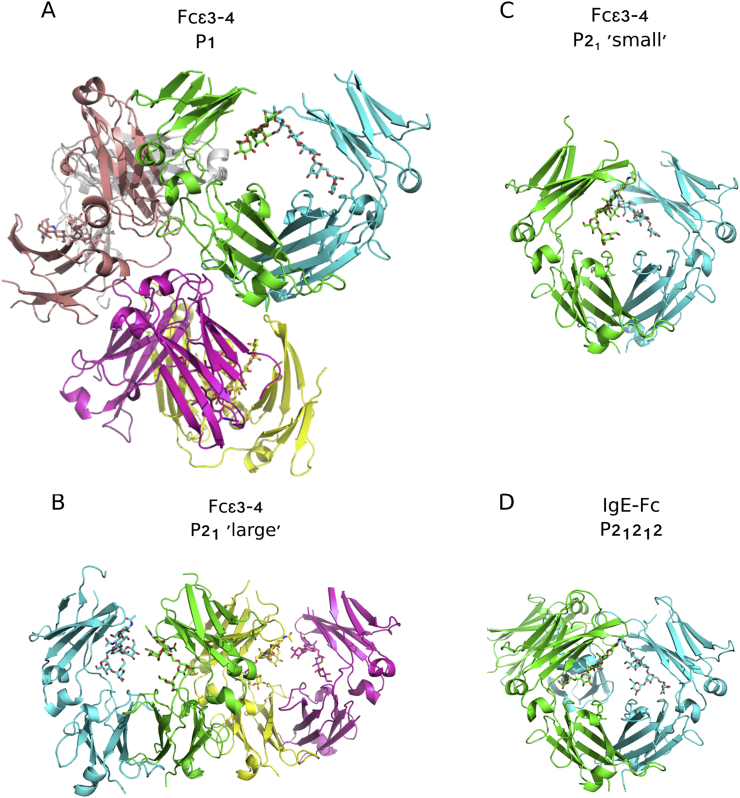
Contents of the asymmetric units of the four crystal forms. Fcε3–4 structures in the P1, P2_1_ ‘large’ and P2_1_ ‘small’ crystal forms and IgE-Fc structure in the P2_1_2_1_2 crystal form with 3, 2, 1 and 1 molecule(s) in each asymmetric unit, respectively. (A) Fcε3–4 P1 crystal form: chain A (cyan), chain B (green), chain C (magenta), chain D (yellow), chain E (salmon) and chain F (grey). (B) Fcε3–4 P2_1_ ‘large’ crystal form: chain A (cyan), chain B (green), chain C (magenta) and chain D (yellow). (C) Fcε3–4 P2_1_ ‘small’ crystal form: chain A (cyan) and chain B (green). (D) IgE-Fc P2_1_2_1_2 crystal form: chain A (cyan) and chain B (green). Carbohydrate is displayed as sticks and modelled in all chains.

The new crystal forms of Fcε3–4, P1 and P2_1_ ‘small’ contain three molecules (six chains) in the asymmetric unit (solved at 2.2 Å resolution), and one molecule (two chains) in the asymmetric unit (solved at 2.26 Å) respectively. They therefore offer four additional and unique packing environments for analysis of the intrinsic flexibility of this molecule (Fig. 1A and C). The other crystal form, P2_1_ ‘large’, with two molecules (four chains) in the asymmetric unit, has been reported earlier [18] but is now solved at 2.0 Å resolution, the highest yet reported for any Fcε3–4 structure (Fig. 1B). The IgE-Fc structure, space group P2_1_2_1_2, with one molecule (two chains) in the asymmetric unit has been determined to 1.75 Å resolution (Fig. 1D), which is the highest reported for this structure. The structure of the carbohydrate, disposition of domains compared with other IgE-Fc and Fcε3–4 structures, and relative flexibility within and between domains, will be discussed in the following sections.

### 3.2 Conformation of the N-linked carbohydrate

**Fig. 2:**
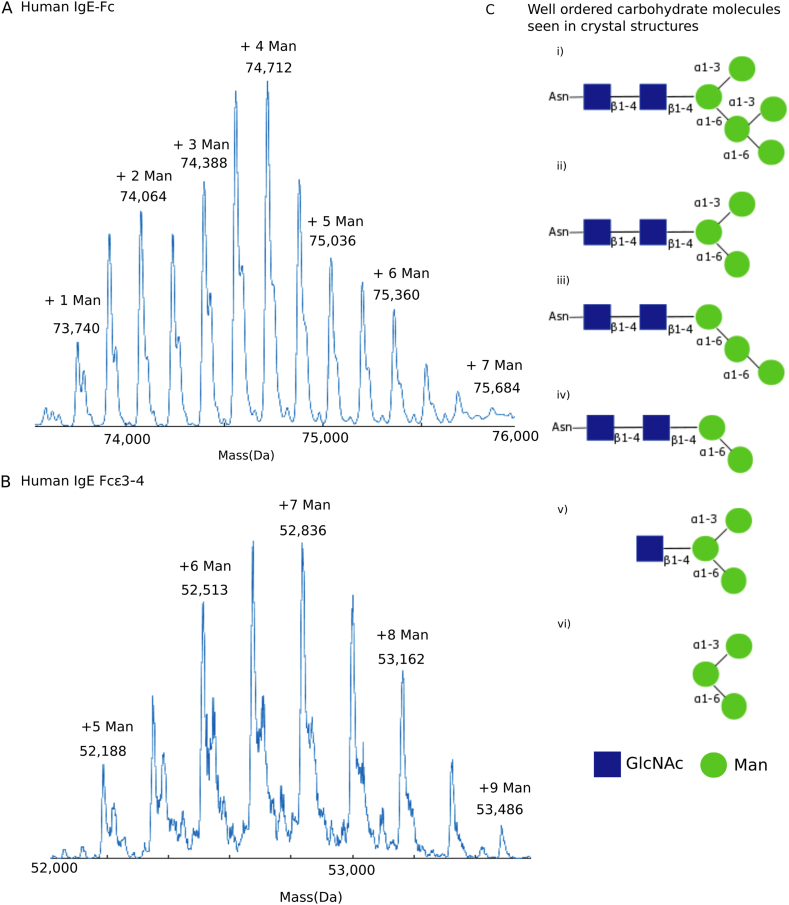
Carbohydrate structures in the four crystal forms. (A) Mass spectrometry trace showing carbohydrate present in IgE-Fc. The molecular mass of the polypeptide dimer is 72,604 Da, and with two *N*-acetylglucosamine (GlcNAc) residues per chain is 73,416 Da. The first peak in the spectrum corresponds to one additional mannose (Man) residue in each chain. (B) Mass spectrometry trace showing carbohydrate present in Fcε3–4. The molecular mass of the polypeptide dimer is 49,756 Da, and with two GlcNac residues per chain is 50,568 Da. (C) Blue squares represent GlcNAc and green circles, Man units. i) Shows the arrangement of the high-mannose N-linked carbohydrate modelled in IgE-Fc chain B, Fcε3–4 P2_1_ ‘large’ chains B and D, and Fcε3–4 P1 chain D. ii) Shows the carbohydrate modelled in Fcε3–4 P2_1_ ‘small’ chains A and B, and in Fcε3–4 P1 chains C and E. iii) Shows the carbohydrate modelled in IgE-Fc chain A and Fcε3–4 P1 chain B. iv) Shows the carbohydrate modelled in Fcε3–4 P2_1_ ‘large’ chain A. v) Shows the carbohydrate modelled in Fcε3–4 P2_1_ ‘large’ chain C and Fcε3–4 P1 chain A. vi) Shows the carbohydrate modelled in Fcε3–4 P1 chain F.

A branched, bi- or tri-antennary, high-mannose type carbohydrate chain ((GlcNAc)_n_(Man)_n_) is N-linked to Asn394 in each Cε3 domain, as seen in previous crystal structures of IgE-Fc and Fcε3–4 at different resolutions, and with varying degrees of disorder. There is heterogeneity in the overall mannose content and in the length of the branches in any population of IgE-Fc or Fcε3–4 molecules (Fig. 2) [12], [13], [14], [15]. Fig. 2C shows the various high-mannose carbohydrate structures seen in the four crystal structures reported. Ordered carbohydrate residues were modelled as follows: IgE-Fc structure, GlcNAc_2_Man_3_ (chain A) (Fig. 2Ciii) and GlcNAc_2_Man_5_ (chain B) (Fig. 2Ci); Fcε3–4 P2_1_ ‘small’ structure, GlcNAc_2_Man_3_ (chains A and B) (Fig. 2Cii); Fcε3–4 P2_1_ ‘large’ structure, GlcNAc_2_Man_5_ (chains B and D) (Fig. 2Ci), GlcNAc_2_Man_2_ (chain A) (Fig. 2Civ) and GlcNAc_1_Man_3_ (chain C) (Fig. 2Cv); Fcε3–4 P1 structure, GlcNAc_2_Man_3_ (chains C and E) (Fig. 2Cii), GlcNAc_2_Man_5_ (chain D) (Fig. 2Ci), GlcNAc_2_Man_3_ (chain B) (Fig. 2Ciii), GlcNAc_1_Man_3_ (chain A) (Fig. 2Cv) and GlcNAc_0_Man_3_ (chain F) (Fig. 2Cvi).

**Fig. 3:**
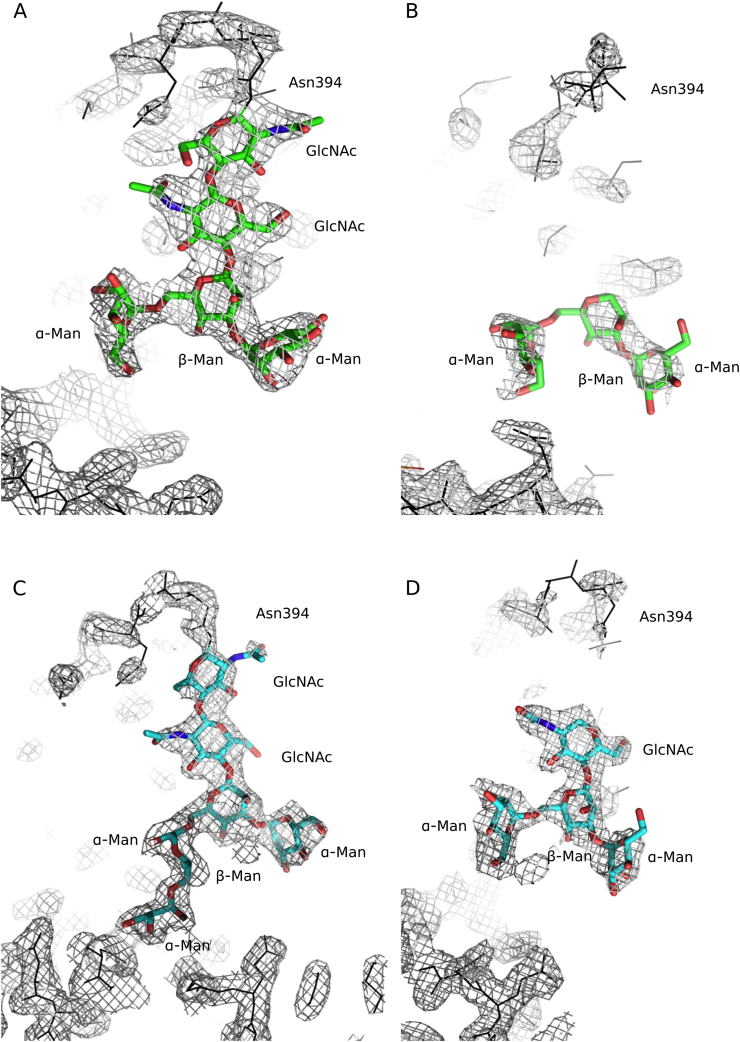
Electron density for carbohydrate at Asn394 in chains E and F of the Fcε3–4 P1 crystal form, and chains C and D of the Fcε3–4 P2_1_ ‘large’ crystal form. (A) Chain E of the Fcε3–4 P1 form: the covalent connection between Asn394 and GlcNAc has well defined density. (B) Chain F of the Fcε3–4 P1 form: Asn394 has poorly defined density and the two GlcNAc units are missing. (C) Chain C of the Fcε3–4 P2_1_ ‘large’ form: the covalent connection between Asn394 and GlcNAc has well defined density. (D) Chain D of the P2_1_ ‘large’ form: Asn394 has poorly defined density and the first GlcNAc unit is missing. Polypeptide is shown as black lines and carbohydrate is coloured by atom type. The electron density is represented by grey mesh (2Fo-Fc map at a contour level of 1σ).

A remarkable feature of the carbohydrate attached to chain D in the Fcε3–4 P2_1_ ‘large’ crystal form and chain F in the Fcε3–4 P1 crystal form is that while the terminal units are well defined, there is poor (or absent) electron density at a contour level of 1σ in the 2FoFc map for the first GlcNAc (P2_1_ ‘large’) or first two GlcNAc units (P1), as well as for the covalent connection between these and Asn394 (Fig. 3B and D). This covalent connection is well defined however in chain D of the Fcε3–4 P2_1_ ‘large’ structure, and chain E of the Fcε3–4 P1 structure (Fig. 3A and C).

**Fig. 4:**
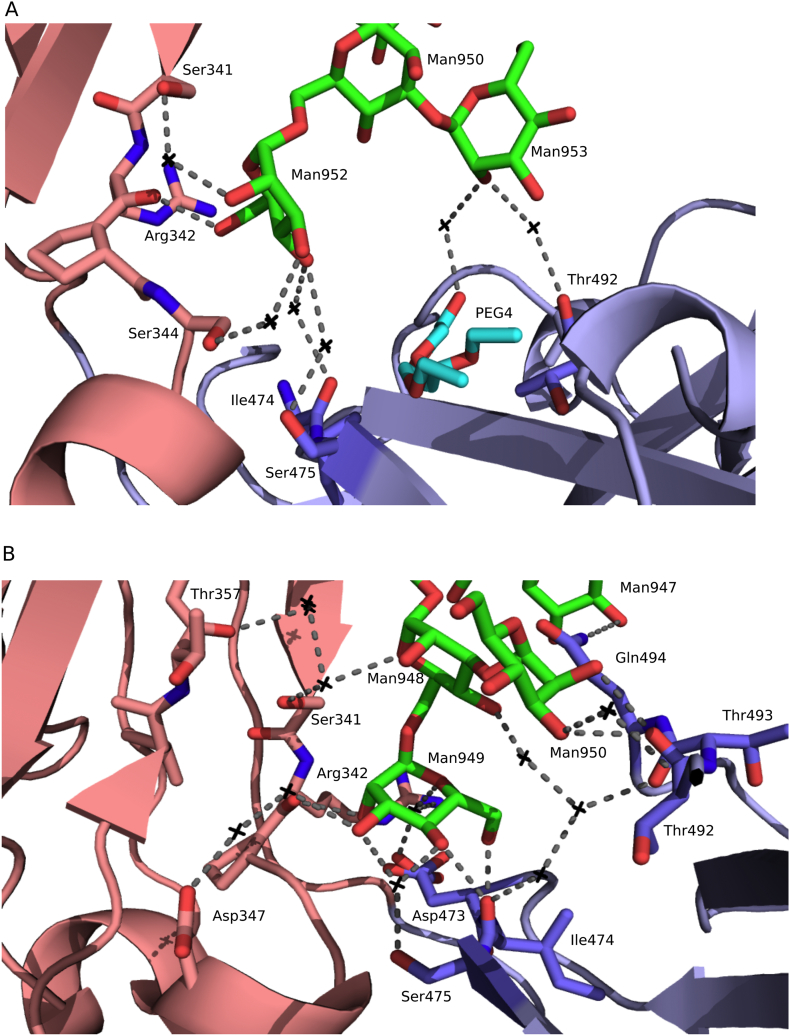
Contacts between carbohydrate and the Cε3 and Cε4 domains in the IgE-Fc crystal structure chain B and the Fcε3–4 P2_1_ ‘large’ crystal form chain D. (A) IgE-Fc crystal structure chain B showing interactions between: Ser341, a water molecule and Man952; Arg342 and Man952; Ser 344, a water molecule and Man952; Ser475, a water molecule and Man952; Ile474, a water molecule and Man952; a polyethylene glycol molecule (PEG4), a water molecule and Man953; Thr492, a water molecule and Man953. (B) Fcε3–4 P2_1_ ‘large’ crystal form chain D showing interactions between: Thr357, three water molecules and Man948; Ser341, a water molecule and Man948; Arg342, a water molecule and Man949; Arg342 and Man949; Asp347, two water molecules and Man949; Asp473 a water molecule and Man949; Ser475 a water molecule and Man949; Ile474 and Man949; Ile474, three water molecules and Man948; Thr492 and Man950; Thr492, a water molecule and Man950; Thr493, two water molecules and Man948; Gln494 and Man947. Hydrogen bonds are shown as grey dotted lines and the black crosses are ordered, bridging water molecules. Carbohydrate residues (shown as sticks) are coloured by atom type, and the Cε3 and Cε4 domains are coloured pink and blue, respectively.

The terminal units make clearly defined contacts with residues of the Cε3 and Cε4 domains. For example, in IgE-Fc chain B a terminal mannose residue, Man 952, forms a hydrogen bond with the main-chain of Arg342. Hydrogen bonds mediated by water molecules are also formed between: Man952 and Ser344, Ser341, Ile474 and Ser475; Man953 and Thr492 (Fig. 4A). In the Fcε3–4 P2_1_ ‘large’ structure chain D, a terminal mannose residue, Man949, forms hydrogen bonds with main-chain atoms of Arg342 and Ile474; Man950 forms hydrogen bonds with Thr492; Man947 forms hydrogen bonds with Gln494. Hydrogen bonds mediated by water molecules are also formed between: Man948 and Ser341, Thr357, Ile474 and Thr493; Man949 and Arg342, Asp347, Asp473, and Ser475; Man950 and Thr492 (Fig. 4B). With the exception of Man947-Gln494, these hydrogen bonds are not seen in the other chains, or in any of the previously published Fcε3–4 or IgE-Fc structures.

In IgG-Fc, complex-type carbohydrate chains are attached at the structurally homologous Asn297 in Cγ2, but in IgG contact is made only with the Cγ2 domains, and never Cγ3. In IgE, the more extensive high-mannose structures have the capability of reaching the Cε4 domains, and the Fcε3–4 P2_1_ ‘large’ structure reported here shows how the terminal carbohydrate units can be stabilised and contribute to linking, non-covalently, the Cε3 and Cε4 domains in IgE, even when the Asn394 loop to which it is covalently connected is relatively disordered.

### 3.3 Quaternary structural comparisons

**Fig. 5:**
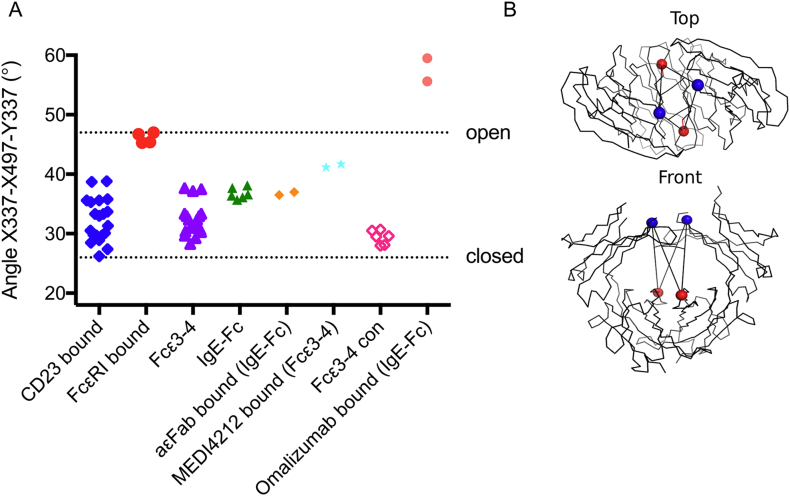
Quaternary structural differences in the Cε3 domains of Fcε3–4 and IgE-Fc. (A) These are defined by the angle X337-X497-Y337 or Y337-Y497-X337, where X is one chain and Y is the paired chain. Plotted are values for CD23-bound structures (blue), FcεRI-bound IgE-Fc and Fcε3–4 structures (red), Fcε3–4 structures, including P1 and P2_1_ ‘large’ and ‘small’ structures reported here (lilac), IgE-Fc, including the structure reported here (green), aεFab-bound IgE-Fc (orange), MEDI4212-bound Fcε3–4 (cyan) artificially constrained Fcε3–4 structures (pink), omalizumab-bound IgE-Fc (salmon). (B) Top and front views of Fcε3–4 indicate residues 337 (in blue) and 497 (in red). The structure illustrated is that of the P2_1_ ‘small’ crystal form.

In their earlier analysis of the quaternary structural variation in IgE-Fc and Fcε3–4, Wurzburg and Jardetzky defined two motions of the Cε3 domains relative to each other, an “opening/closing” and a “swinging” [18]. The Cε4 domain pair is virtually identical in all structures and provides a reference point for comparing the disposition of the Cε3 domains. These authors defined two distance measurements: Cα of residue 394 of one chain (X) to Cα497 of the other chain (Y) for the “opening/closing”, and Cα336(X) to Cα336(Y) for the “swinging”. These were displayed as a 2d plot [18]. As these are in fact one concerted movement, with more “closed” structures showing less “swing” and more “open” structures showing more “swing” we propose combining both components with a single measurement of the angle defined by Cα337(X)-Cα497(X)-Cα337(Y) (Fig. 5), using the descriptors “open” and “closed”. Residue 337 was selected as it is located within the Cε3 domain, away from the Cε2–Cε3 domain linker which undergoes a conformational change when IgE-Fc unbends [23].

We have compared all available IgE-Fc and Fcε3–4 structures (now 34 molecules, compared with only 10 in the earlier analysis [18]); the single parameter clearly describes the wide range of open and closed Cε3 conformations (Fig. 5). The complexes with FcεRI, and IgE-Fc bound to the omalizumab Fab, are the most open, while those bound to CD23 and the numerous uncomplexed Fcε3–4 structures show how they almost span the entire range from closed to the open conformation in the FcεRI complex: none however is as open as that seen in the omalizumab Fab/IgE-Fc complex. In contrast to Fcε3–4, there is to date only a single crystal form of uncomplexed IgE-Fc despite extensive crystallisation trials (authors' unpublished observations). This may imply that the Cε2 domains stabilise, through extensive contact with one of the Cε3 domains, an intermediate quaternary structure (between open and closed) [17]. The recent structure of the complex between IgE-Fc and two anti-IgE aεFab molecules unexpectedly revealed that the Cε2 domains can also adopt an extended, linear conformation with respect to the Fcε3–4 region, with no Cε2/Cε3 domain contact [23]. When this occurs, the Cε3 domains adopt a quaternary structure that is incompatible with FcεRI binding; the quaternary structure lies between the two extremes (Fig. 5). A similar phenomenon was observed for Fcε3–4 bound to another anti-IgE Fab, MEDI4212 [24]. The artificially constrained Fcε3–4 structures and complexes with DARPin and omalizumab [19], [20], [25], are clearly more closed (Fig. 5). The Cε3 domains in this Fcε3–4 construct are, however, tethered by an artificial disulphide bond that prevents any relative movement. In contrast, IgE-Fc bound to omalizumab exhibits the most open conformation seen, and is also incompatible with FcεRI binding [26]; IgE-Fc is partially bent in this complex. It is also clear from this comparison that despite the 26 independent chain conformations observed in free Fcε3–4 (and the single uncomplexed IgE-Fc structure), none is sufficiently open to permit FcεRI binding; engagement of receptor must occur at one sub-site before full opening occurs. In contrast, many of the free Fcε3–4 structures adopt conformations that are receptive for CD23 binding.

### 3.4 Flexibility of the Cε3 domains

**Fig. 6:**
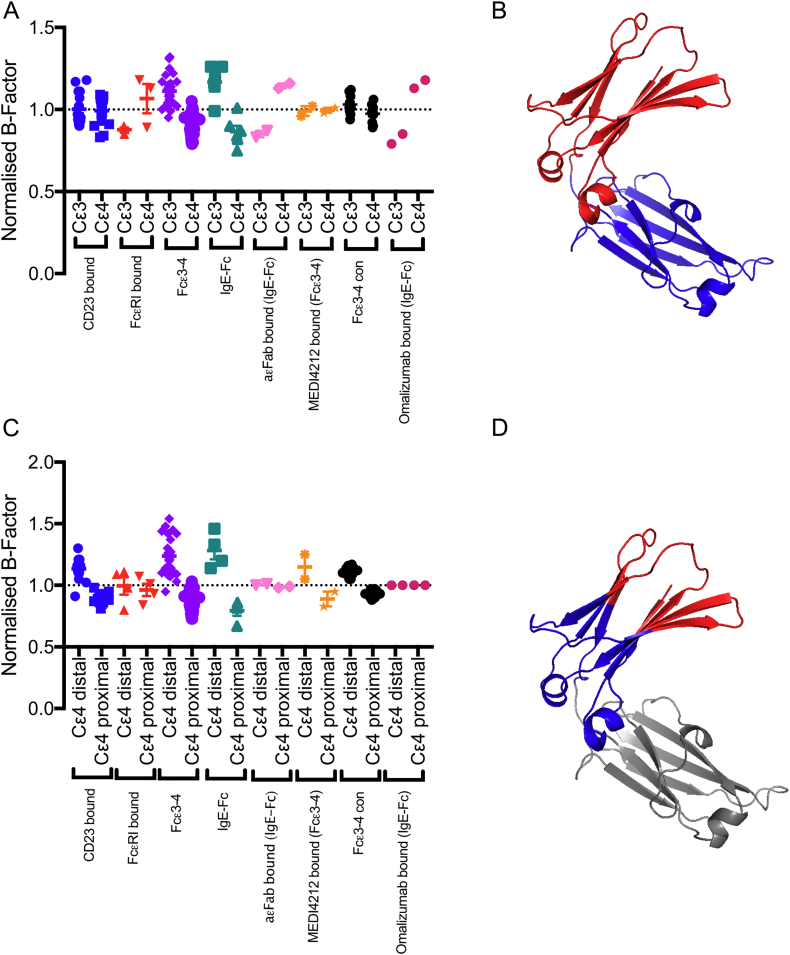
B-factor analysis of Fcε3–4 between domains and within Cε3. (A) Plot of normalised B-factors (refer Materials and methods) for Cε3 and Cε4 domains: CD23-bound structures (blue), FcεRI-bound structures (red), Fcε3–4 structures (lilac), IgE-Fc structure (teal), aεFab-bound (extended) IgE-Fc (pink), MEDI4212-bound Fcε3–4 (orange), artificially constrained Fcε3–4 structures (black) and omalizumab-bound IgE-Fc (dark pink). (B) Indicates the Cε3 (red) and Cε4 (blue) domains. (C) Graph of normalised B-factors for Cε4-distal and Cε4-proximal regions of the Cε3 domains: colours as for panel A. (D) Indicates the Cε4-distal (red) and Cε4-proximal (blue) regions of the Cε3 domains. The Cε4 domains are coloured grey.

The new structures show disorder in the loops and adjoining β-strands of the region of Cε3 most distant from the Cε4 domains. (The two halves of the Cε3 domain will here be referred to as the Cε4-proximal and Cε4-distal regions; for definition by residue number, refer to Materials and methods). While this has been seen in other Fcε3–4 (and also some IgG-Fc structures [43]), it is particularly marked in the new Fcε3–4 P1 form, and this prompted an analysis of the relative B-factors across all of the Fcε3–4 and IgE-Fc structures, comparing Cε3 with Cε4 (Fig. 6A&B), and also the Cε4-proximal and Cε4-distal regions of Cε3 (Fig. 6C&D). In order to compare B-factors from structures refined with different protocols and at different resolutions, the values for each chain or domain were normalised by dividing by the average B-factor (refer to Materials and methods).

The B-factor analysis revealed that the Cε3 domains showed greater intrinsic flexibility than the Cε4 domains in the unbound Fcε3–4 and IgE-Fc structures, despite the range of different crystal packing environments (Fig. 6A). In the FcεRI-bound, aεFab-bound and omalizumab Fab-bound IgE-Fc structures, the opposite effect is seen, undoubtedly due to the stabilising effect of FcεRI, aεFab and omalizumab Fab binding to the Cε3 domains. In the CD23-bound, MEDI4212-bound and artificially constrained Fcε3–4 crystal structures, B-factors for the Cε3 and Cε4 domains are similar (Fig. 6A).

Comparison of B-factors *within* the Cε3 domain (Fig. 6B) shows that the Cε4-distal region displays the greater degree of disorder or flexibility in virtually all of the structures, including IgE-Fc and also the Fcε3–4/CD23 complexes. The Cε4 domains, through extensive interactions with Cε3, stabilise the Cε4-proximal regions of Cε3, but the Cε2 domains in IgE-Fc do not have the same effect upon the Cε4-distal region (Fig. 6A). In the FcεRI-bound, the extended aεFab-bound and the omalizumab Fab-bound IgE-Fc structures, the Cε4-distal region of Cε3 is stabilised, unsurprisingly, since this is the location of the two receptor sub-sites, one in each Cε3, and the aεFab binding site [4], [5]; the omalizumab Fab epitope is located on the exposed outer face of the Cε3 domain [26]. The pattern for MEDI4212-bound Fcε3–4 is again different, but its epitope principally involves the Cε4-proximal region of Cε3.

This flexibility of the Cε3 domains in the context of IgE-Fc and Fcε3–4 and their stabilisation by FcεRI-binding is consistent with the more extreme behaviour of the Cε3 domain when studied in isolation: alone it behaves as a partially folded “molten globule” [7], [8], [9], [10], yet it can bind sFcεRIα [7], [11] and, in its presence, adopt a more folded structure [8], [9].

### 3.5 Differential stability of the IgE-Fc domains

**Fig. 7:**
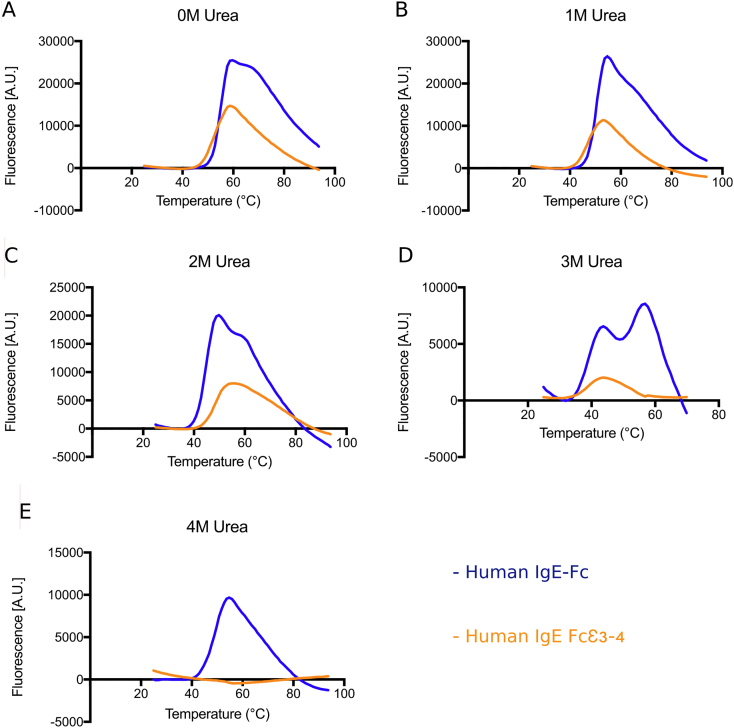
Thermal stability of IgE-Fc and Fcε3–4. The unfolding of IgE-Fc and Fcε3–4 as a function of temperature and urea concentration, measured by DSF, are compared. (A) 0 M urea. (B) 1 M urea. (C) 2 M urea. (D) 3 M urea. (E) 4 M urea. Melting temperatures reported in Table 2 were determined from the first derivative plots calculated from these curves.

The thermally-induced unfolding of Fcε3–4 and IgE-Fc in the presence of increasing concentrations of urea was measured by DSF (Fig. 7). The Fcε3–4 data show a single unfolding event at all urea concentrations. The unfolding event occurs at 52 °C in the absence of urea, and at lower melting temperatures as the urea concentration is increased (Fig. 7, Table 2). This implies that the Cε3 and Cε4 domains unfold cooperatively. The IgE-Fc data however shows a two-state unfolding (Fig. 7), the first event, in the absence of urea, at 55 °C (T_m_1) and the second at 64 °C (T_m_2). The latter is taken to be the unfolding of the Cε2 domains, occurring after that of Cε3 and Cε4. Both T_m_1 and T_m_2 decrease with increasing urea concentration (Fig. 7, Table 2). For Fcε3–4, and also for these domains within IgE-Fc, 4 M urea causes complete unfolding in the absence of heating, whereas > 6 M urea is required to achieve this for the Cε2 domains.

The intensity of the signals from Fcε3–4 are consistently smaller than those from IgE-Fc (at the same concentration), which may be due to differences in the aggregation properties of the two proteins as denaturation occurs. Aggregation reduces the hydrophobic surface area to which Sypro-orange molecules can bind, thus reducing the signal. The Cε3 domains, and in particular the N-terminal regions, are more flexible (as seen in crystal structures) and perhaps inherently more prone to unfolding in Fcε3–4 compared with IgE-Fc, thus promoting aggregation of the former. However, as seen in the B-factor analysis (Fig. 6A), the stabilising effect of the (Cε2)_2_ domain pair upon the Fcε3–4 region is relatively modest, since the T_m_ and T_m_1 values for Fcε3–4 and IgE-Fc respectively are very similar under all urea conditions (Table 2).

## 4 Conclusions

The Cε3 domains are central to the function of IgE since they contain the receptor-binding sites for both FcεRI and CD23. These two sites are located at opposite ends of the Cε3 domain, yet their binding is mutually incompatible [6], [28]. Allosteric communication between these two sites clearly involves quaternary structural changes in IgE-Fc and Fcε3–4 domains, predominantly in the relative disposition of the Cε3 domains [6], [28]. However, the Cε3 domain has been identified as an outlier according to various measures of folding and stability [10], and it may be that this unique behaviour is required in order to transmit the allosteric signal. The three new Fcε3–4 crystal structures reported here contribute to understanding both the intrinsic flexibility of the Cε3 domains and their quaternary structure, and we have now compared all of the (34) available IgE-Fc and Fcε3–4 structures. This highlights the more extreme conformational variation in Cε3 than was seen in an earlier analysis based upon the far fewer structures then available [18]. Furthermore, the enhanced resolution for two of the structures reported here provides new detail concerning the ordered regions of the N-linked carbohydrate that is attached to this intrinsically flexible domain.

Analysis of the B-factors from all the published crystal structures together with the new structures presented here confirms the enhanced intrinsic flexibility of the Cε3 domains and demonstrates that this is most pronounced in their Cε4-distal regions. The ability to average over so many different structures in different crystal forms, overcomes any local effects of crystal packing constraints. The Cε3 domains are stabilised by FcεRI binding (Fig. 6), consistent with the results of experiments conducted with isolated Cε3 domains binding to sFcεRIα [8], [9] and with thermal stability data reported for IgE-Fc with and without sFcεRIα [44].

The thermal stability data that we present here implicate the Cε3 domains as the most susceptible to denaturation within IgE-Fc. The thermal lability of IgE has been recognised for a long time. Even before the recognition of IgE as an antibody, the loss of “reaginic” activity in atopic sera by heating to 56 °C was reported [29], and shortly after the discovery of the IgE antibody class, this unusual thermal susceptibility compared with IgG (for which T_m_ values are typically ~ 72 °C), was confirmed [30]. Furthermore, on the basis of circular dichroism spectroscopy and retention or loss of antigenic determinants as a function of temperature, Dorrington and Bennich were able to write that “the irreversible changes seen with intact IgE result from structural changes in that portion of chain C-terminal to Fc”-ε [their terminology for the Cε2 domains]”, *i.e.* in the Cε3–4 domains [30]. We show here that IgE-Fc unfolds in a two-step process, the first involving the Fcε3–4 region with T_m_ values similar to that of intact IgE, and the second involving the Cε2 domains. Since the T_m_1 and T_m_ values for IgE-Fc and Fcε3–4 respectively are very similar, the Cε2 domains have only a marginal influence on the thermal stability of the Cε3 domains.

In IgG-Fc, glycosylation of the complex type, N-linked at the structurally homologous Asn297 in each Cγ2, contributes to the thermal stability of this domain, and the two domains have distinct T_m_ values: glycosylated, 71 °C and 82 °C for Cγ2 and Cγ3 respectively; fully deglycosylated, 66 °C and 82 °C [45]. In contrast, Fcε3–4 unfolds cooperatively at the much lower temperature of 52 °C even when fully glycosylated (Fig. 7 and Table 2). Deglycosylation of Fcε3–4 further reduces the T_m_ by 4 °C [46]. The inability of the high-mannose carbohydrate to stabilise the Cε3 domain is consistent not only with its intrinsic lability, but also with the remarkable observation that at the point of covalent connection to Asn394 in Cε3, in chain C of the Fcε3–4 P2_1_ “large” form and chain F of the P1 form, the flexibility and/or disorder is so great that there is no visible electron density (Fig. 3B&D); this is despite the fact that, as seen most clearly in the structure reported here, well-ordered carbohydrate units make contact with Cε4 (Fig. 4). In IgG-Fc, no carbohydrate contact is ever made with the Cγ3 domains. This difference between IgG and IgE is also reflected in the functional requirement for Fc glycosylation. Whereas substantially reduced glycosylation compromises IgG-Fc receptor-mediated effector functions [47], glycosylation at Asn394, while required for folding and secretion of IgE *in vitro* and *in vivo* [15], is not essential for either FcεRI or CD23 binding, since refolded IgE-Fc lacking carbohydrate altogether has native-like affinity for both receptors [48], [49], [50], [51]. The relationship between glycosylation, structure and function in IgE is thus very different to IgG, and appears to be yet another unique feature of this class of antibody.

## Accession numbers

Coordinates and structure factors have been deposited in the Protein Data Bank with accession numbers: Fcε3–4 P1, PDB ID: 5MOI; Fcε3–4 P2_1_ ‘small’, PDB ID: 5MOJ; Fcε3–4 P2_1_ ‘large’, PDB ID: 5MOK; IgE-Fc, PDB ID: 5MOL.

## Transparency document

Transparency document. Image 2
